# Supplementary material for: The Effect of Applied Potential on the Li-mediated Nitrogen Reduction Reaction Performance
Source: Nat Commun. 2025 Nov 27;16:10635. doi: 10.1038/s41467-025-65627-w (PMC12660919; doi:10.1038/s41467-025-65627-w)
Supplement: Supplementary file 1 — Supplementary Information [file 41467_2025_65627_MOESM1_ESM.docx]

# Supplementary Information (SI) to

# The Effect of Applied Potential on the Li-mediated Nitrogen Reduction Reaction Performance

Boaz Izelaar ^1^, Pranav Karanth ^2^, Arash Toghraei ^3^, Santosh K. Pal ^2^, Nandalal Girichandran ^1^, Mark Weijers ^2^, Ruud W. A. Hendrikx ^4^, Fokko M. Mulder ^2^, Ruud Kortlever ^1,^*

^1^ Process and Energy Department, Faculty of Mechanical Engineering, Delft University of Technology, 2628 CB Delft, The Netherlands

^2^ Chemical Engineering Department, Faculty of Applied Sciences, Delft University of Technology, 2629 HZ Delft, The Netherlands

^3^ Énergie, Matériaux, Télécommunications Research Centre, Institute National de la Recherche Scientifique (INRS), 1650 Bd. Lionel-Boulet, Varennes, Quebec J3X 1P7, Canada

^4^ Material Science and Engineering Department, Faculty of Mechanical Engineering, Delft University of Technology, 2628 CB Delft, The Netherlands

*Corresponding Author; E-mail: [R.Kortlever@tudelft.nl](mailto:R.Kortlever@tudelft.nl)

## Supplementary Figures


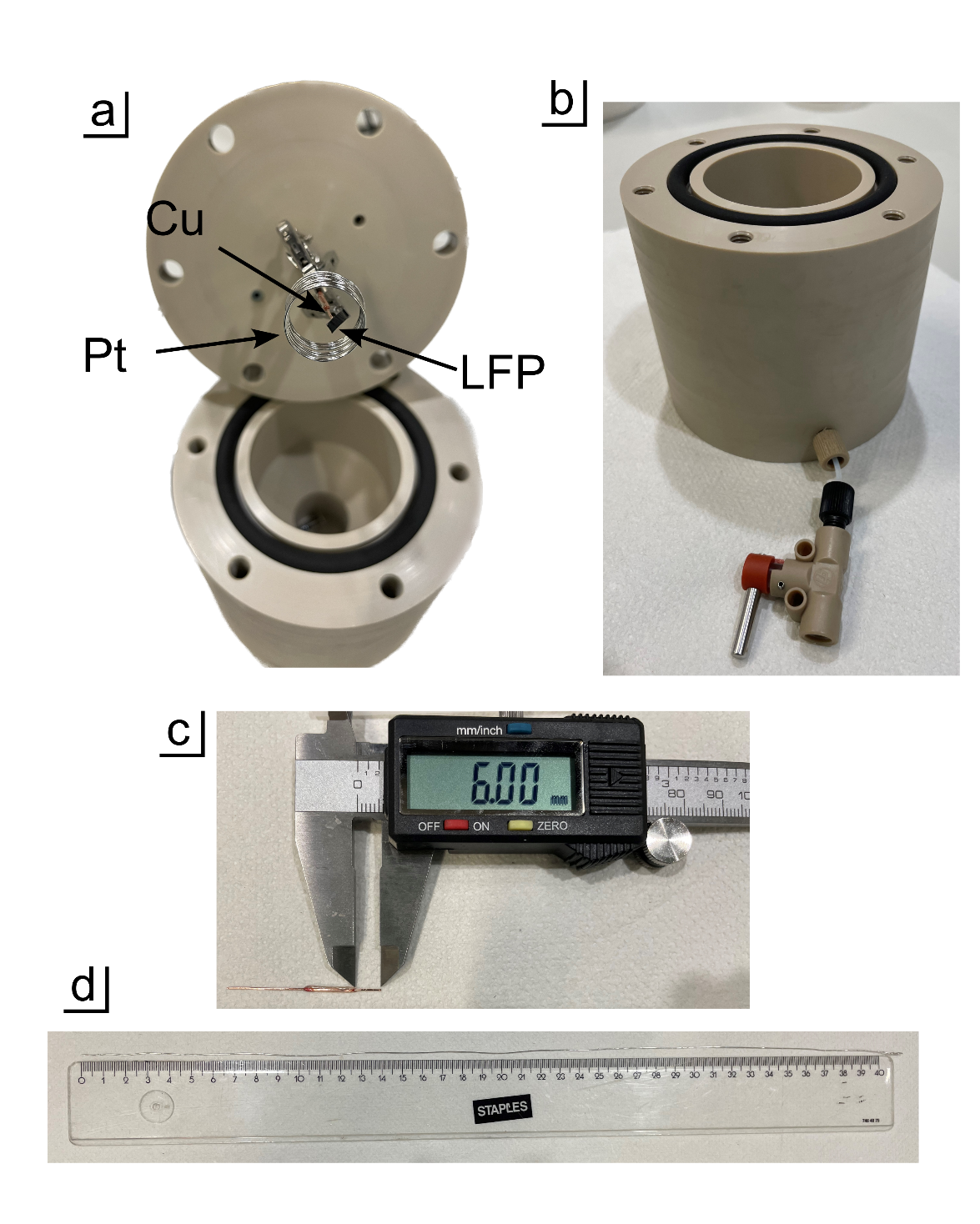


**Supplementary Fig. 1. Photographs of the autoclave cell configuration.** (a) Home-built autoclave three-electrode cell configuration. (b) Modified cell body with a drain to remove electrolyte before degassing. (c) Glass isolated Cu wire with Ø0.5mm and 6 mm length (A = 0.1 cm^2^). (d) Pt wire has a Ø0.5mm and 40 cm length which is eventually reshaped into a Ø1.4 cm coil (A ≈ 6.3 cm^2^). Typically, half of the coiled Pt wire was submerged in the electrolyte.


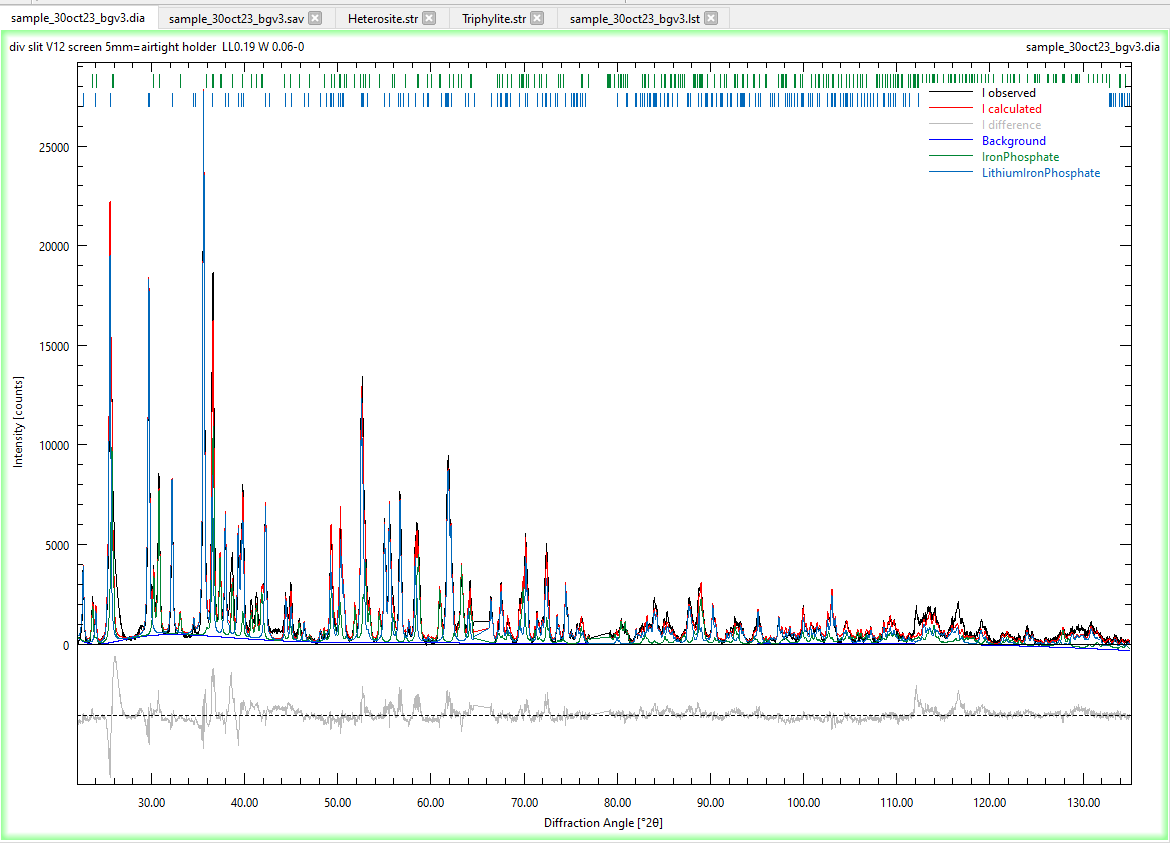


**Supplementary Fig. 2. Rietveld refinement fitting of the XRD pattern of a partially delithiated LFP sheet in the Profex software environment.** Corresponding to Figure 2c from the main manuscript. Reference XRD patterns of LiFePO_4_ (04-011-8634) and FePO_4_ (04-011-8635) were taken from the ICDD pdf4 database. The phase composition is 67 mol% LiFePO_4_ and 33 mol% FePO_4_.


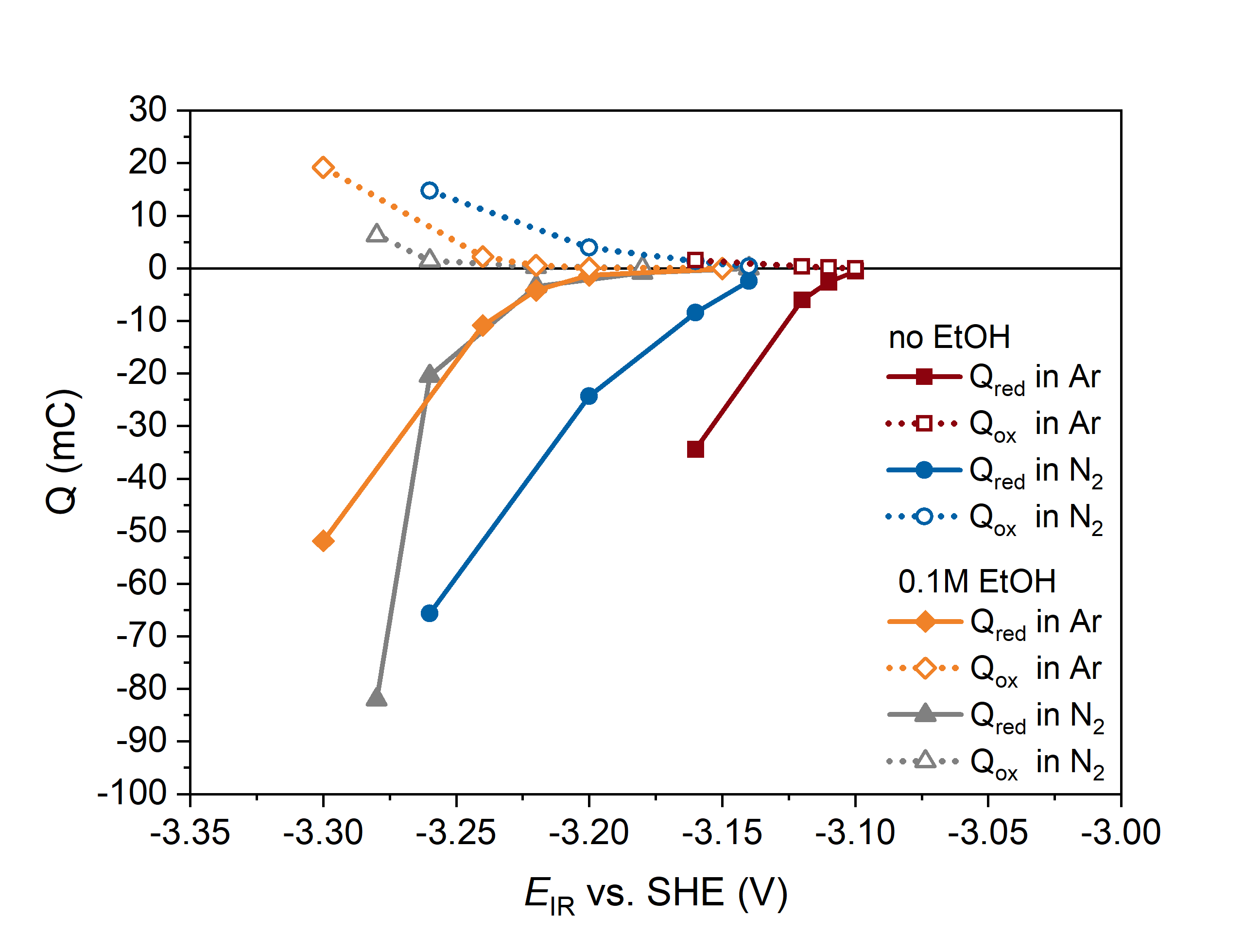


**Supplementary Fig. 3. Total charge of the reduction and oxidation peaks of the cyclic voltammograms.** Corresponding to Figure 3 of the main manuscript. Base electrolyte is 2 M LiTFSI in THF with or without 0.1 M EtOH. Potential is for 85% *IR*_u_ compensated by the build-in software in EC-Lab.

**Supplementary Fig. 4. Cyclic voltammetry while introducing N_2_ gas.** Li^+^ reduction peaks in the cyclic voltammograms are changing when releasing 2.5 bar N_2_ pressure into the autoclave cell after the first scan. The first scan is a voltammetry under Ar which is comparable with Figure 2c from the main manuscript. The scan rate (υ) is 20 mV s^-1^ and the electrolyte is 2 M LiTFSI in 0.1 M EtOH/THF. The potential is for 85% *IR*_u_ compensated by the build-in software in EC-Lab.

**Supplementary Fig. 5. An example of cyclic voltammograms before and after a 4-hour chronoamperometry measurement at a certain reduction potential.** The intersection between the reduction and oxidation peaks represents the Li/Li^+^ equilibrium potential at -3.03 V vs. SHE. The equilibrium potential only shifted by 2 mV, indicating that our LFP reference electrode is stable throughout the electrochemical measurement. The measurements were conducted with 2 M LiTFSI dissolved in 0.1 M EtOH/THF at 20 bar N_2_ pressure and room temperature using a scan rate of 20 mV s^-1^. The potential is for 85% *IR*_u_ compensated by the build-in software in EC-Lab.


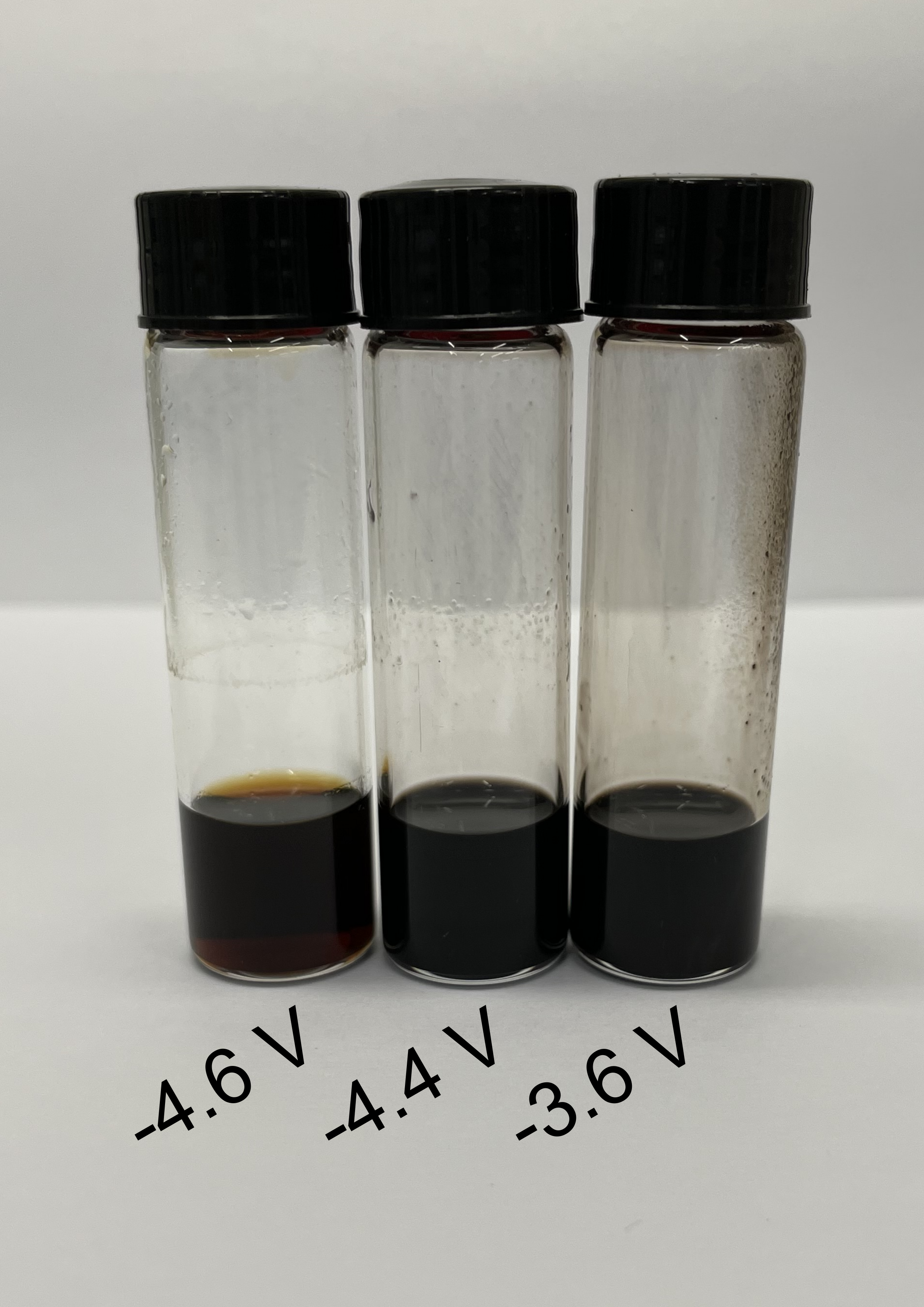


**Supplementary Fig. 6. Photograph of collected electrolyte after a measurement.** The electrolyte color turned black when the SEI fully dissolved into the electrolyte during the N_2_ depressurization step, transfer of the cell into the glovebox or disassembling. The electrolyte was typically transparent at low overpotentials because the SEIs were not excessively thick (see Figure 4 and 5 of the main manuscript).

**Supplementary Fig. 7. Chronoamperometry results showing the accumulated charge, measured anodic potential and the fixed working electrode potential.** (a) -3.1 V vs. SHE, (b), -3.7 V vs. SHE, (c) -4.1 V vs. SHE and (d) -4.6 V vs. SHE. The measurements were conducted with 2 M LiTFSI dissolved in 0.1 M EtOH/THF at 20 bar N_2_ pressure and room temperature.


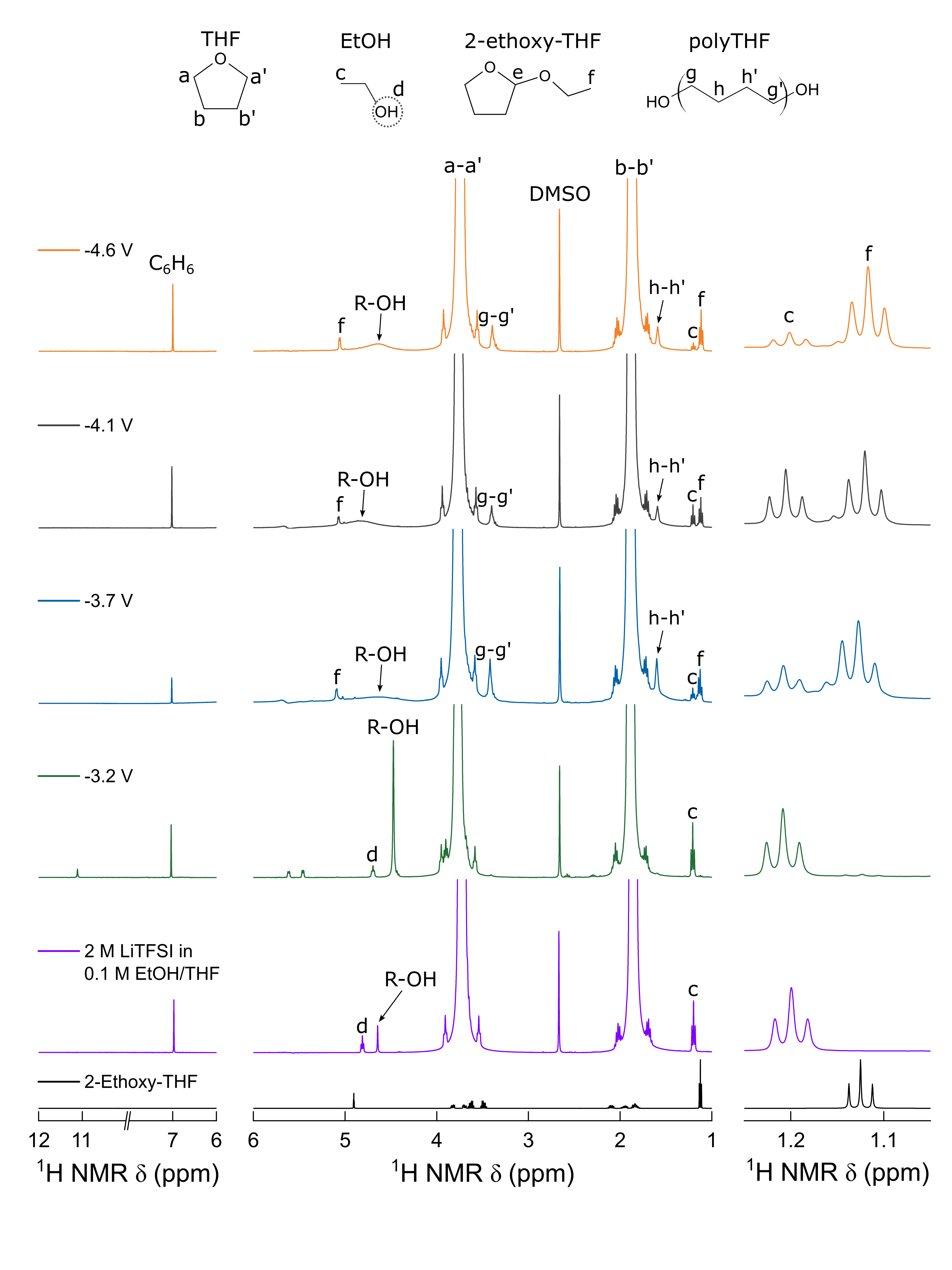


**Supplementary Fig. 8. Liquid ^1^H NMR spectra of the electrolyte before and after chronoamperometry at different applied potentials.** The 2-ethoxy-THF reference spectra was simulated in MestReNova 15.


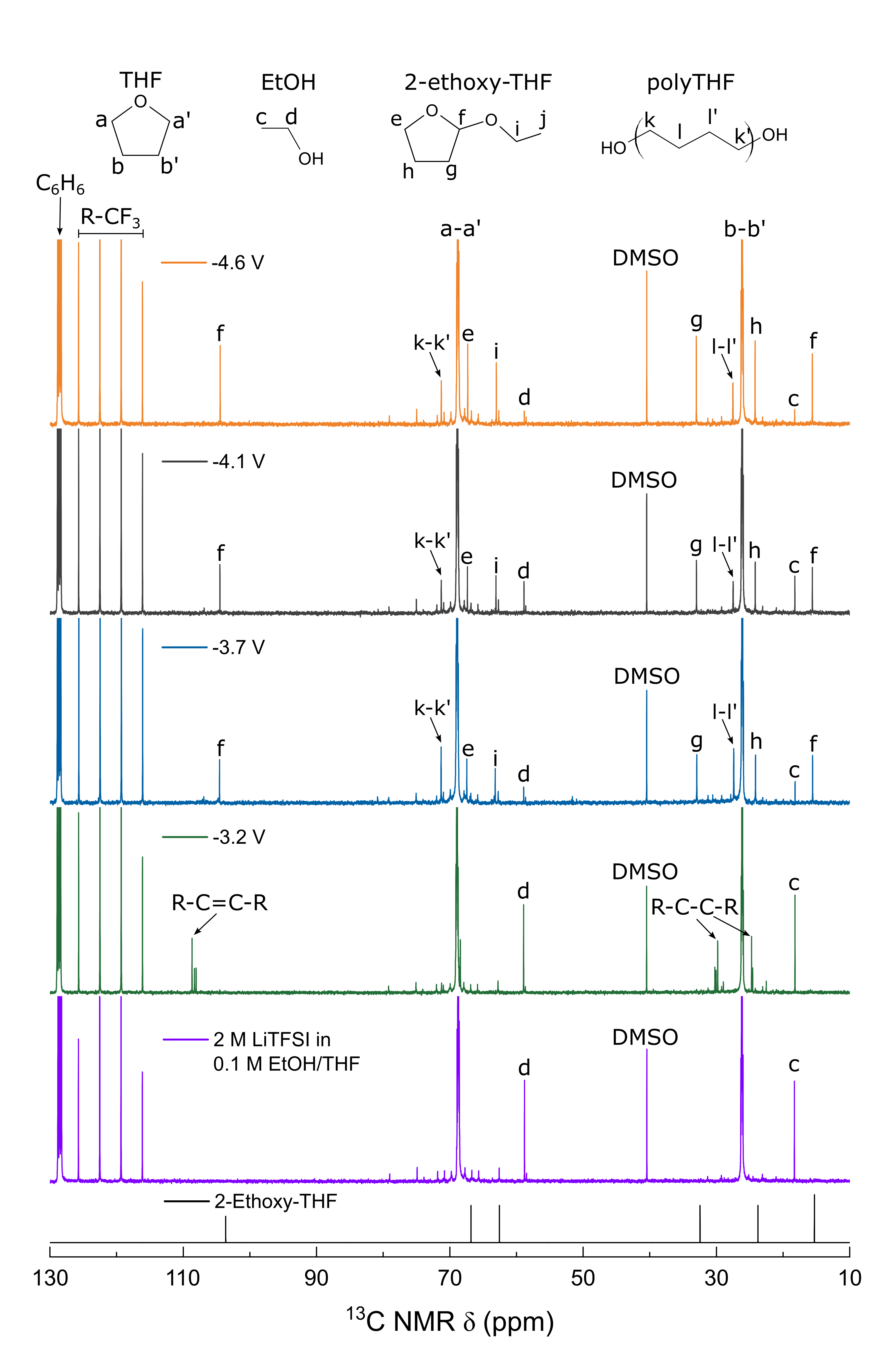
**Supplementary Fig. 9. Liquid ^13^C NMR spectra of the electrolyte before and after chronoamperometry at different applied potentials.** The 2-ethoxy-THF reference spectra was simulated in MestReNova 15.

**Supplementary Fig. 10. Liquid ^19^F NMR spectra of the electrolyte before and after chronoamperometry at different applied potentials.**


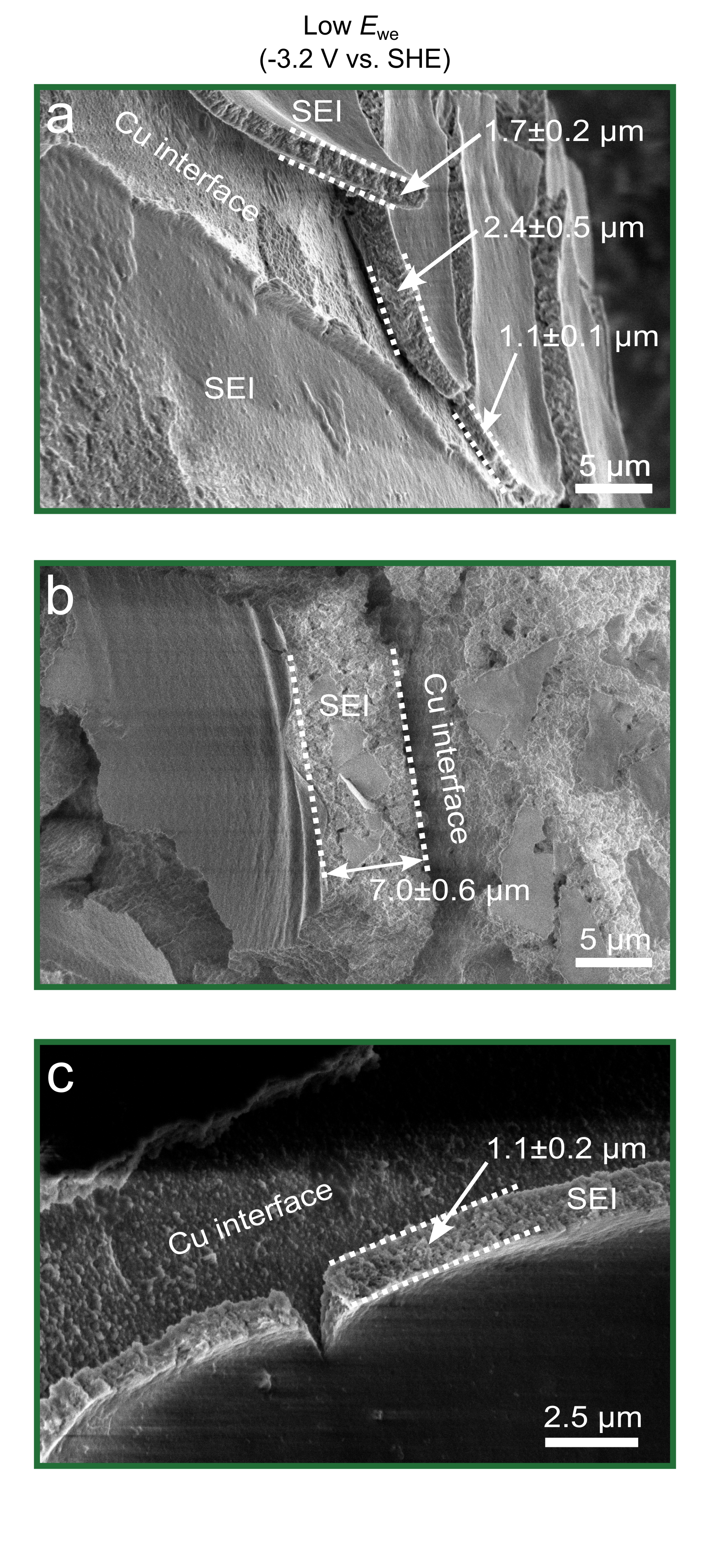


**Supplementary Fig. 11.** **SEM images of the Cu electrode post-measurement at -3.2 V, focusing on the SEI.** (a,b) have a magnification of 5000x and (c) 12000x. The electron beam-induced charging effects are related to the insulating properties of the SEI. The SEI thickness varies between 1-7 μm and can only be used as a rough estimate because the cross-section is poorly defined, and the thickness varies along the length of the SEI. The thickness labels in the figure were obtained by taking the average and the standard deviation of 8 different spots along the dotted line. The length scales were obtained with ImageJ.


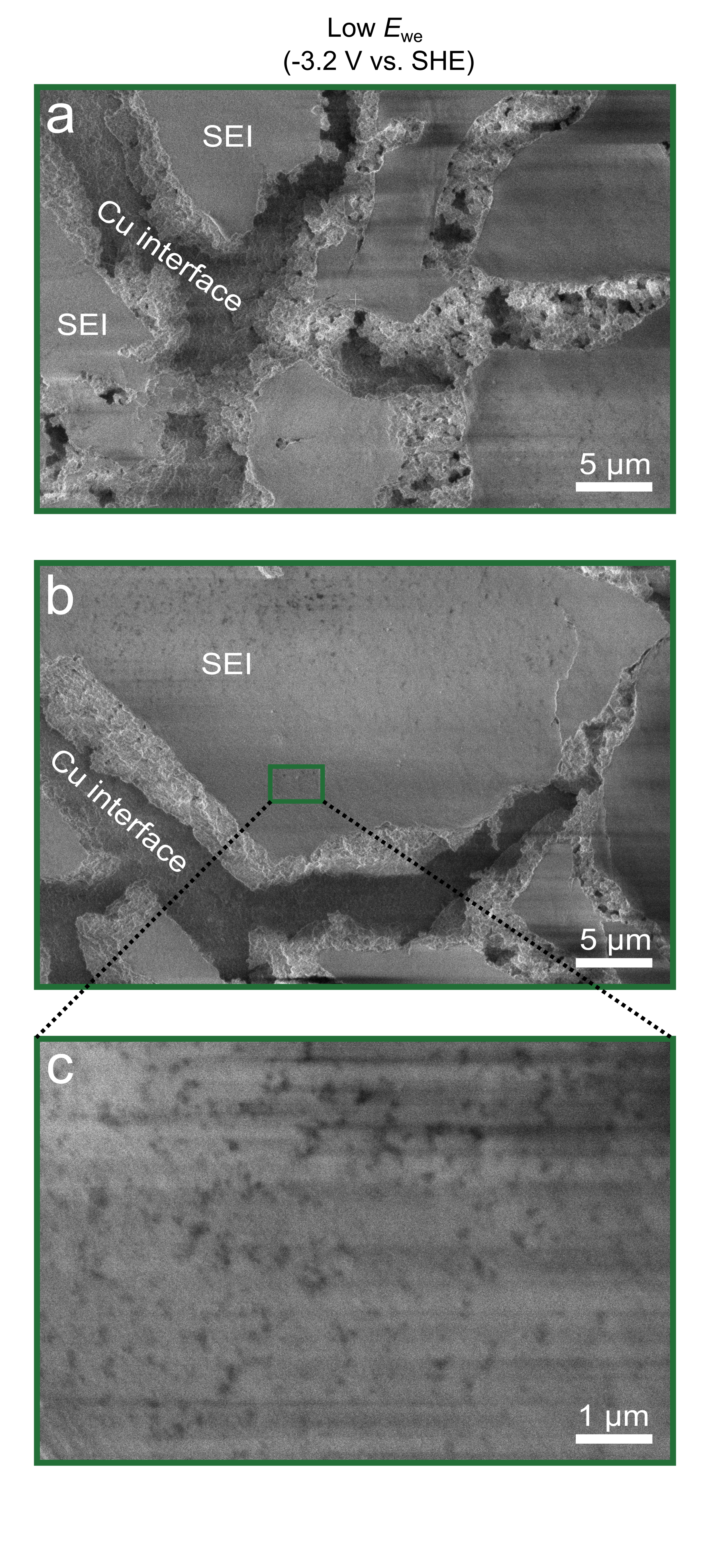


**Supplementary Fig. 12.** **Additional scanning electron microscopy images of the Cu electrode post-measurement at -3.2 V, focusing on the SEI.** (a,b) have a magnification of 5000x and (c) 25000x. The electron beam-induced charging effects are related to the insulating properties of the SEI.


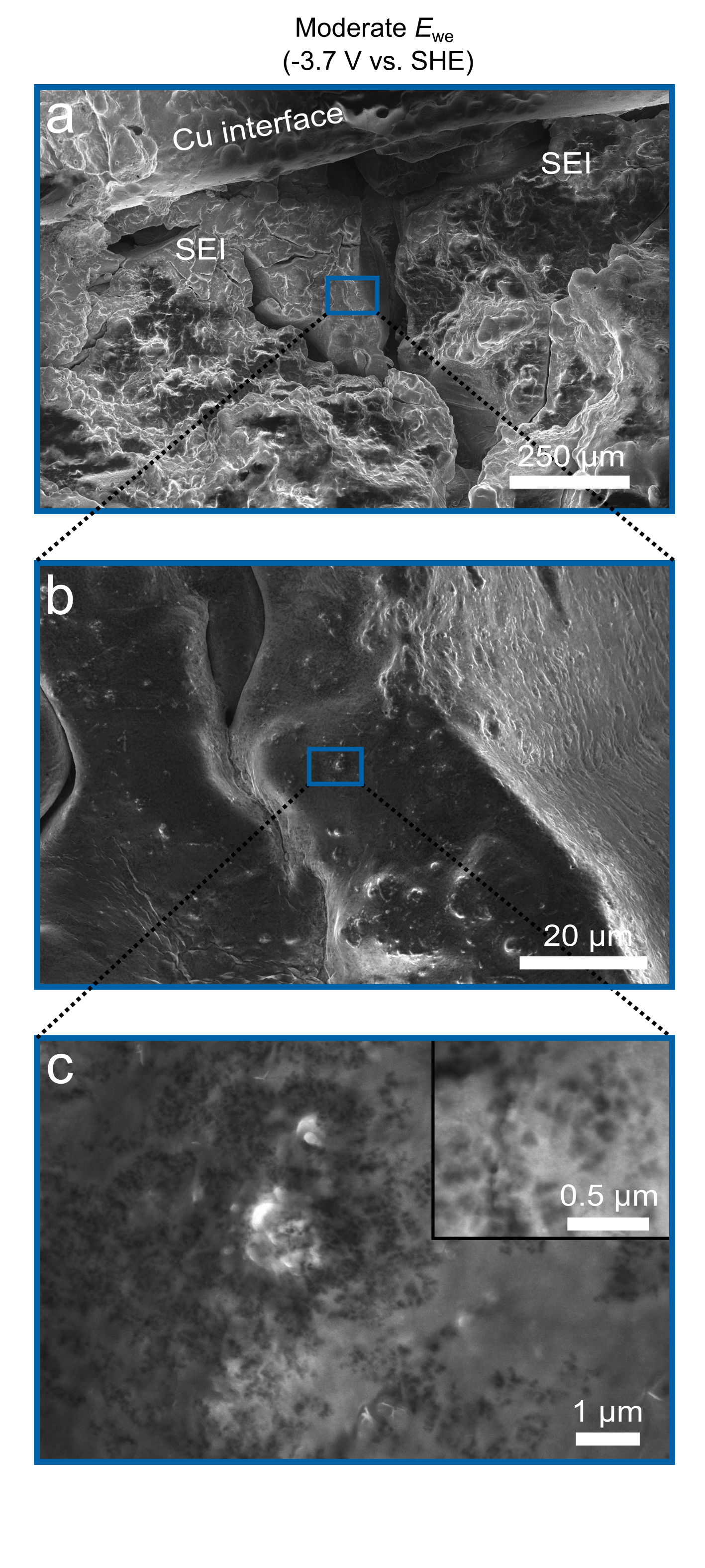


**Supplementary Fig. 13.** **SEM image of the Cu electrode post-measurement at -3.7 V, focusing on the SEI.** (a) 150x, (b) 2000x, (c) 20000x and 50000x for the inset. Spots with a darker contrast in (c) may indicate nanosized pores in the SEI structure.


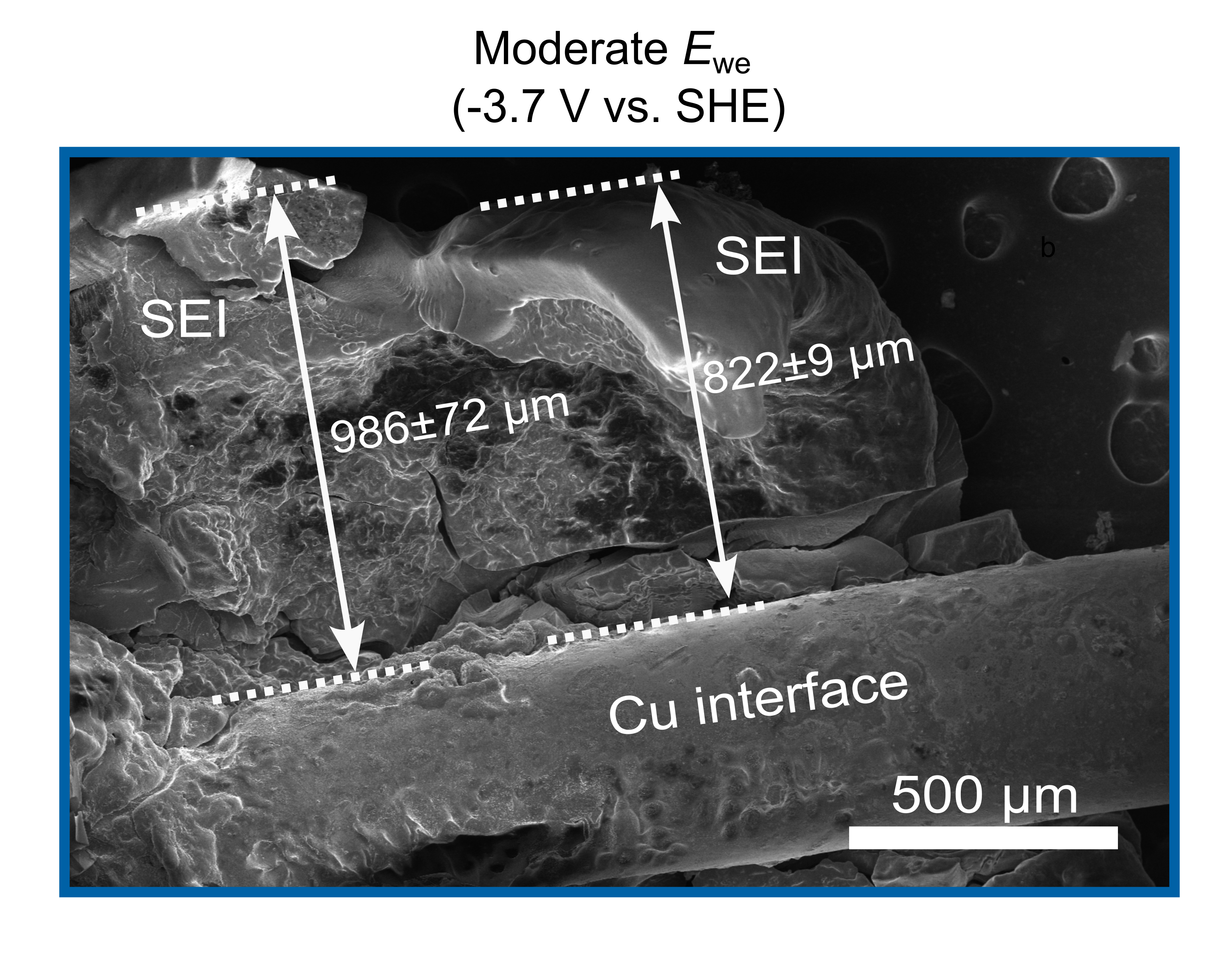


**Supplementary Fig. 14.** **SEM image of the Cu electrode post-measurement at -3.7 V, focusing on the SEI cross-section at 100 x magnification.** The SEI thickness varies between 0.8-1 mm, which can be only be used as a rough estimate because the cross-section is poorly defined, and the thickness varies along the length of the Cu wire. The thickness labels in the Figure were obtained by taking the average and the standard deviation of 8 different spots along the dotted line. The length scales were obtained with ImageJ.


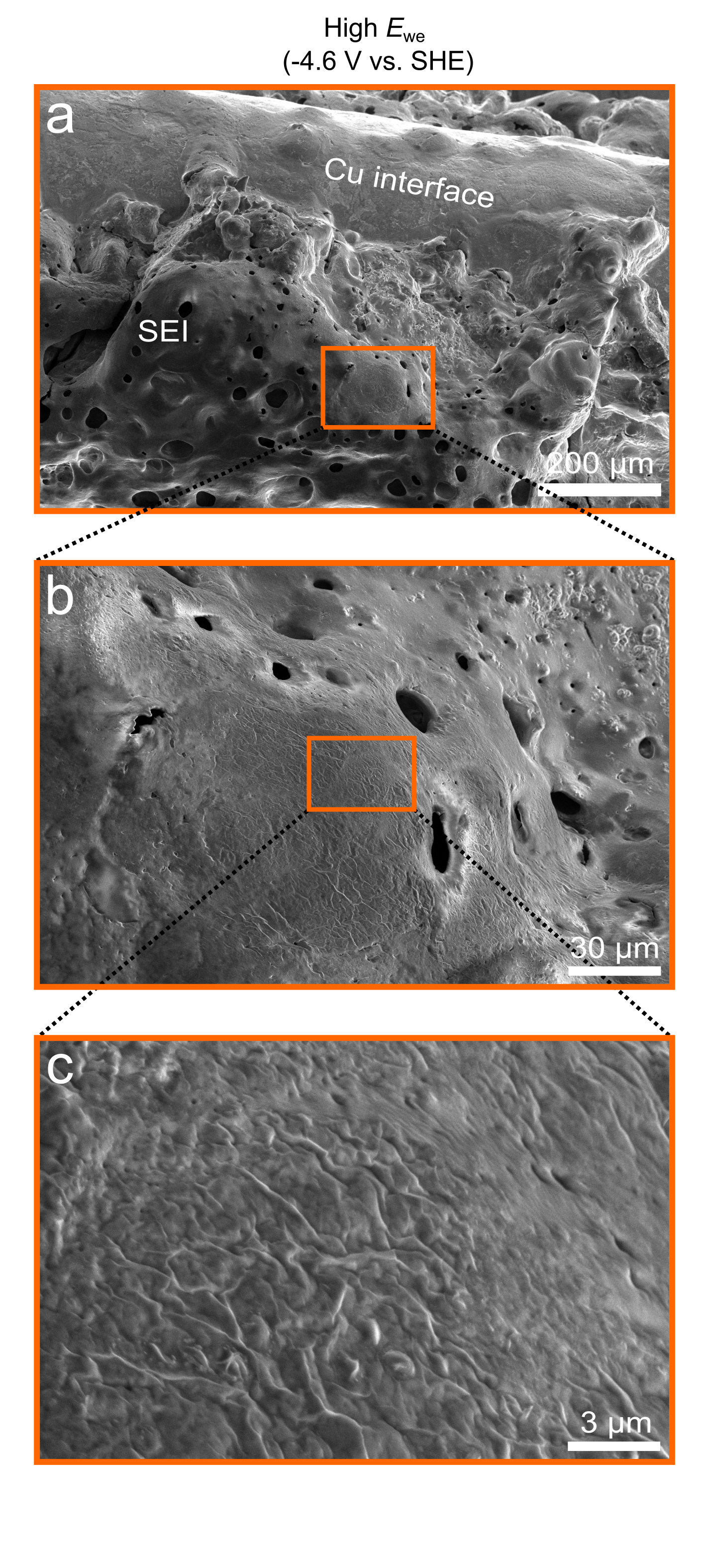


**Supplementary Fig. 15.** **SEM images of the Cu electrode post-measurement at -4.6 V, focusing on the SEI.** (a) 200x, (b) 1000x, (c) 10000x.


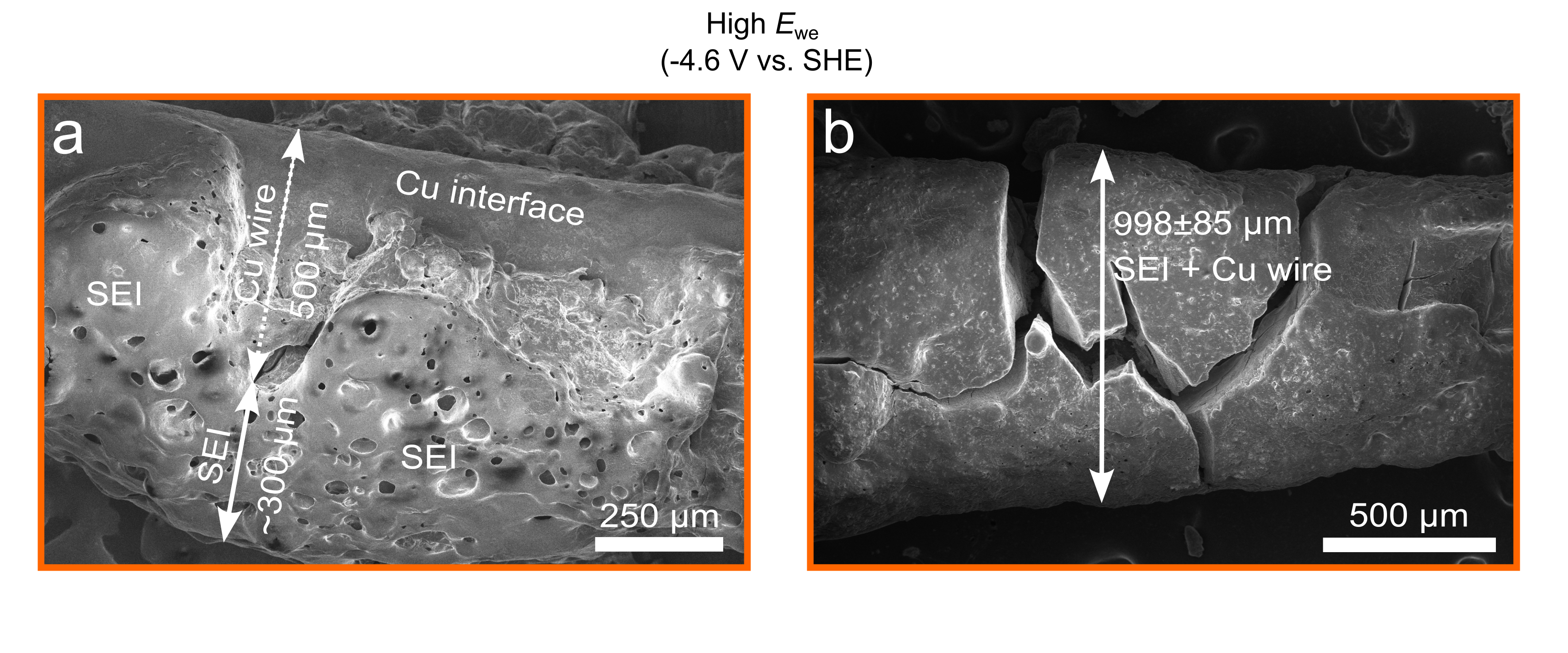


**Supplementary Fig. 16. SEM images of the Cu electrode post-measurement at -4.6 V, focusing on the SEI thickness.** (a) SEM image of the SEI at 150 x magnification. The indicated SEI thickness of ~300 μm is a rough estimate because the cross-section is poorly defined, and the thickness varies along the length of the Cu wire. (b) SEM image of another SEI covering the Cu electrode at 100 x magnification without cross-section. The thickness of the Cu wire including SEI is ~1 mm (measured with ImageJ). By subtracting the Cu wire thickness (0.5 mm) and dividing it by two, the SEI thickness becomes roughly 250 μm, which is in the same ballpark range as the estimated SEI thickness in (a).

**Supplementary Fig. 17.** **High resolution Li 1s XPS spectra of the SEI obtained after chronoamperometry at -3.2 V, -3.7 V and -4.6 V after 0 s, 60 s, 240 s, 600 s of Ar^+^ etching.** Surface scan of LiTFSI is included for referencing. 2 M LiTFSI in 0.1 M EtOH/THF was used as the electrolyte. **Supplementary Fig. 18.** **XPS survey of the SEI obtained after chronoamperometry at -3.2 V after 0 s, 60 s, 240 s, 600 s of Ar^+^ etching.** 2 M LiTFSI in 0.1 M EtOH/THF was used as the electrolyte.

 **Supplementary Fig. 19.** **XPS survey of the SEI obtained after chronoamperometry at -3.7 V after 0 s, 60 s, 240 s, 600 s of Ar^+^ etching.** 2 M LiTFSI in 0.1 M EtOH/THF was used as the electrolyte.

**Supplementary Fig. 20. XPS survey of the SEI obtained after chronoamperometry at -4.6 V after 0 s, 60 s, 240 s, 600 s of Ar^+^ etching.** 2 M LiTFSI in 0.1 M EtOH/THF was used as the electrolyte.

**Supplementary Fig. 21. Solid-state ^13^C NMR spectra of the SEI obtained post-measurement at (a) -3.7 V and (b) -4.6 V.** 2 M LiTFSI in 0.1 M EtOH/THF was used as the electrolyte. The spectra in the top of each figure indicates a standard ^13^C NMR spectra with ^1^H decoupling, while the bottom spectra represent the results of the ^13^C – ^1^H cross-polarization measurement. ^13^C – ^1^H cross-polarization only includes the ^13^C NMR spectral contribution from the molecules with C-H functionalities. The two sharp peaks at 68.10 ppm and 24.97 ppm match well with the chemical shift of THF. Signal contribution from other organic SEI compounds such as, LiEtO could not be identified. Other peaks in the standard ^13^C NMR spectra are CF_3_ (120.18 ppm) and an interfering signal from KBr used as a filler for the NMR rotor.

**Supplementary Fig. 22. XPS depth profiling of the solid electrolyte interphase** **at 1 M LiTFSI.** (a) Elemental composition of the SEI obtained post-measurement at -4.4 V after 0-600 s of Ar^+^ etching. (b) High resolution XPS spectra of C 1s, F 1s, S 2p, O 1s and Li 1s of the SEI obtained post-measurement at -4.4 V after 0 s, 60 s, 240 s and 600 s of Ar^+^ etching. Vertical dashed lines indicate binding energies of known chemical species. Surface scan of LiTFSI is included for referencing. 1 M LiTFSI in 0.1 M EtOH/THF was used as the electrolyte.

 **Supplementary Fig. 23.** **XPS survey of the SEI obtained after chronoamperomety at -4.4 V after 0 s, 60 s, 240 s, 600 s of Ar^+^ etching.** 1 M LiTFSI in 0.1 M EtOH/THF was used as the electrolyte.

**Supplementary Fig. 24. Raman spectroscopy of different LiTFSI concentrations dissolved in THF.** (a) THF ring breathing vibration (914 cm^-1^) with additional shoulder peaks at 902 cm^-1^ and 922 cm^-1^. (b) THF ring stretching vibration between 1030-1035 cm^-1^.^1^

**Supplementary Fig. 25. Performance data from this work and other literature reports in the Li-NRR field.** The literature entrees from the batch systems (indicated in blue) include an additional energy input for sacrificial THF oxidation. The blue ellipsoidal area represents the spread of our results ranging from -3.1 V to -4.6 V using 2 M LiTFSI in 0.1 M EtOH/THF as electrolyte. For the batch cell literature entrees, we used ref ^2, 3, 4^ at 1 bar, ref ^5^ at 10 bar, ref ^6^ at 15 bar, ref ^7, 8, 9, 10^ at 20 bar and ref ^11, 12, 13, 14^ for the continuous flow cell. All data is summarized in Supplementary Table 2.


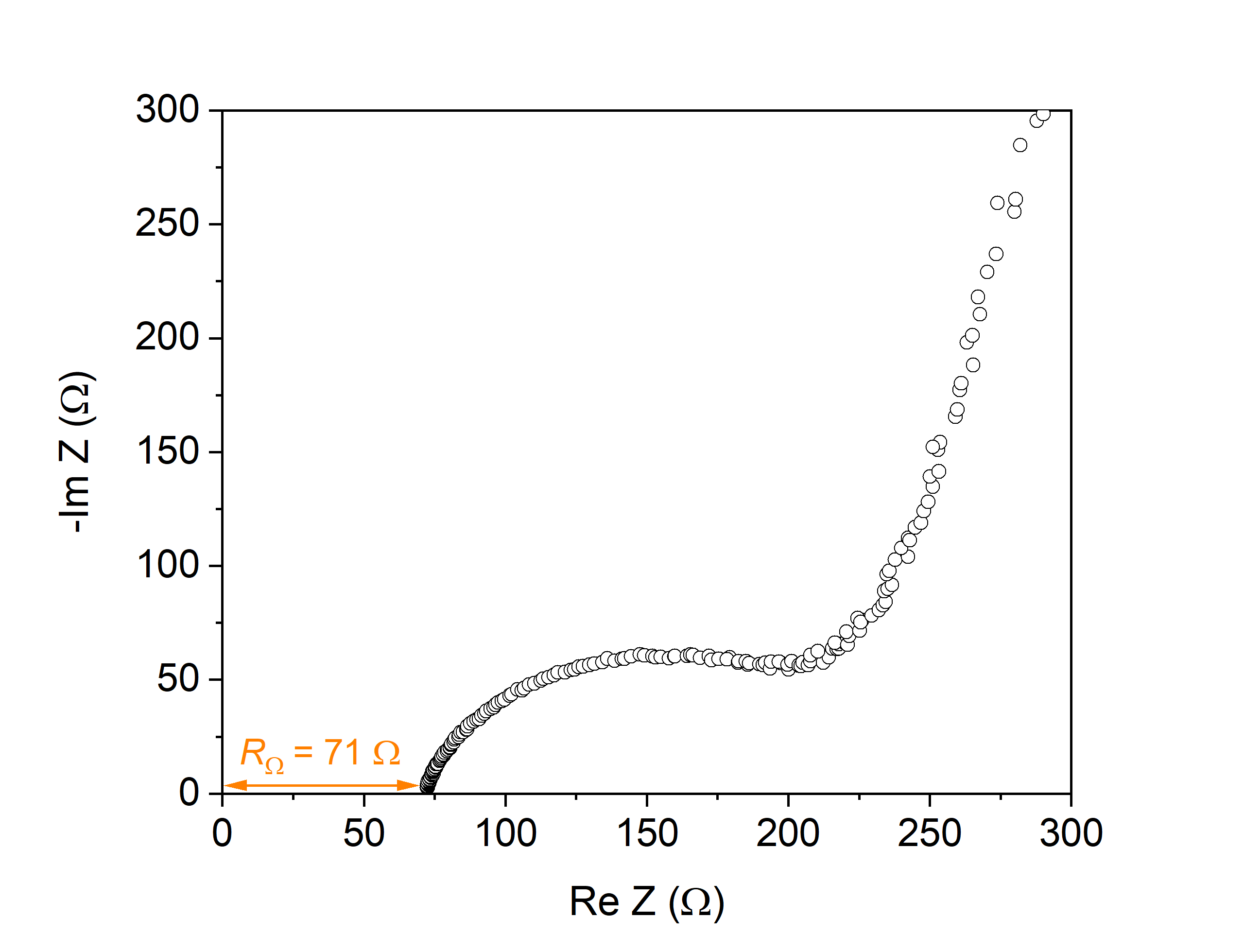


**Supplementary Fig. 26. Nyquist plot of the autoclave cell in a two-electrode configuration.** Cu and Pt were used as working and counter electrode, respectively. 2 M LiTFSI in 0.1 M EtOH/THF was used as the electrolyte. The ohmic resistance (*R*_Ω_) is the impedance contribution at high EIS frequencies and is solely related to the Real Z contribution. *R*_Ω_ can be obtained from the Nyquist plot by taking the graph’s intersection with the Real Z axes, which is equal to 71 Ω. The measurement was conducted at 20 bar N_2_ pressure and room temperature.


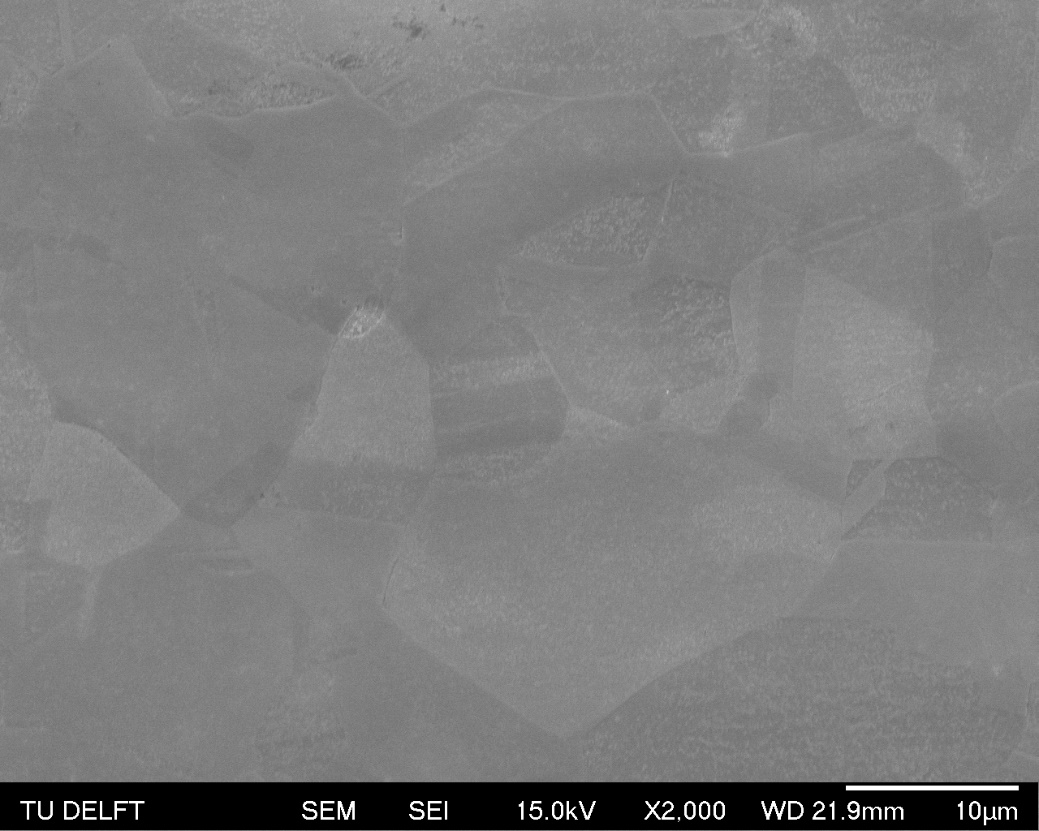

**Supplementary Fig. 27. Scanning electron microscopy image of the Cu electrode after electropolishing.**

**Supplementary Fig. 28. Moisture content in the solvents after drying them over molecular sieves.** The error bars represent the mean ± standard deviation derived from three independent measurements.


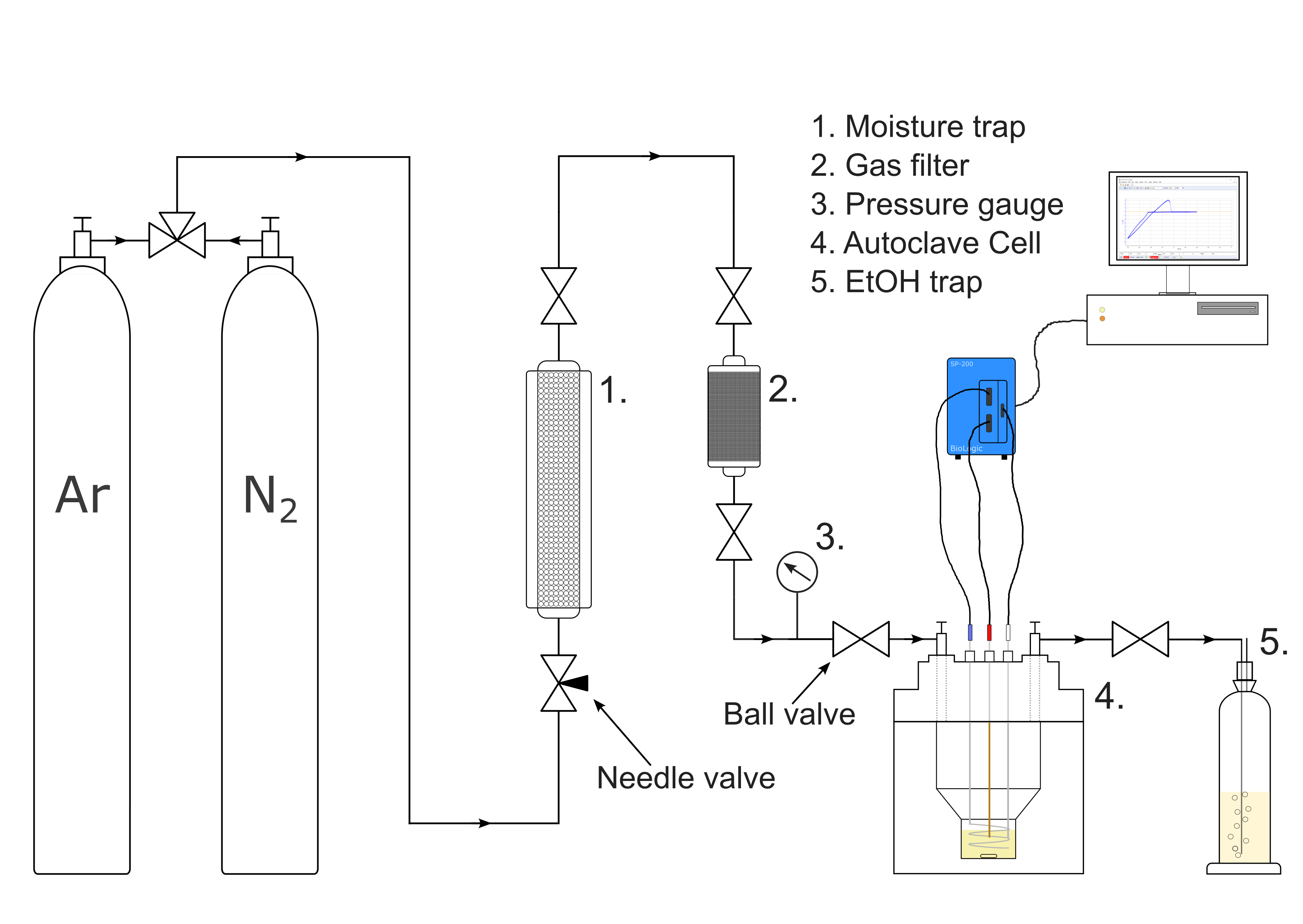


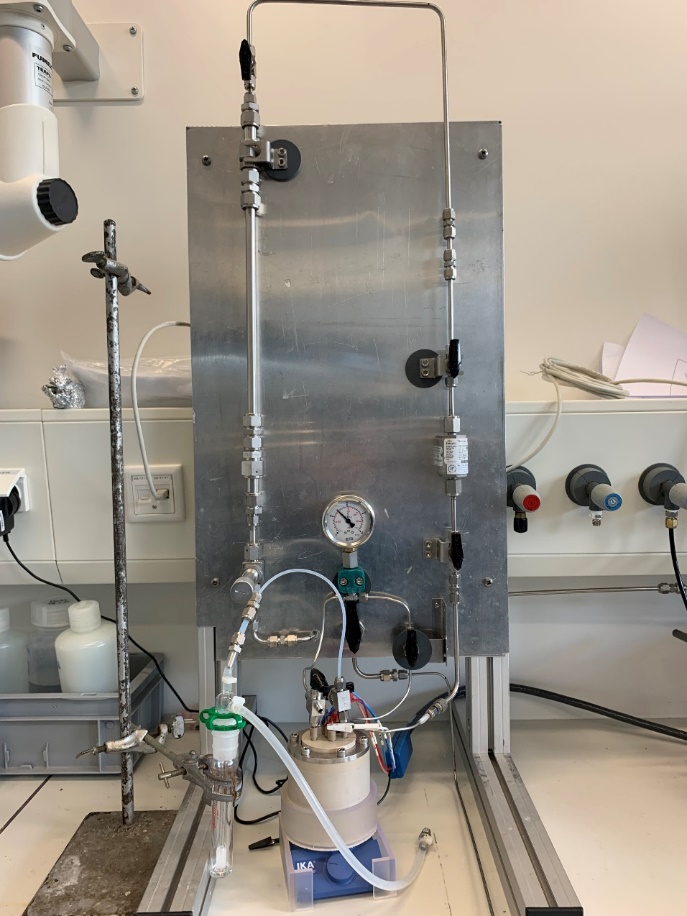


**Supplementary Fig. 29. Schematic (top) and photograph (bottom) of the experimental setup configuration.** These include the gas cleaning skid, cell, stirrer and a bubbler filled with ethanol functioning as an air lock during depressurization. A local suction point was always placed over the top of the cell during operation but was omitted for the photograph.


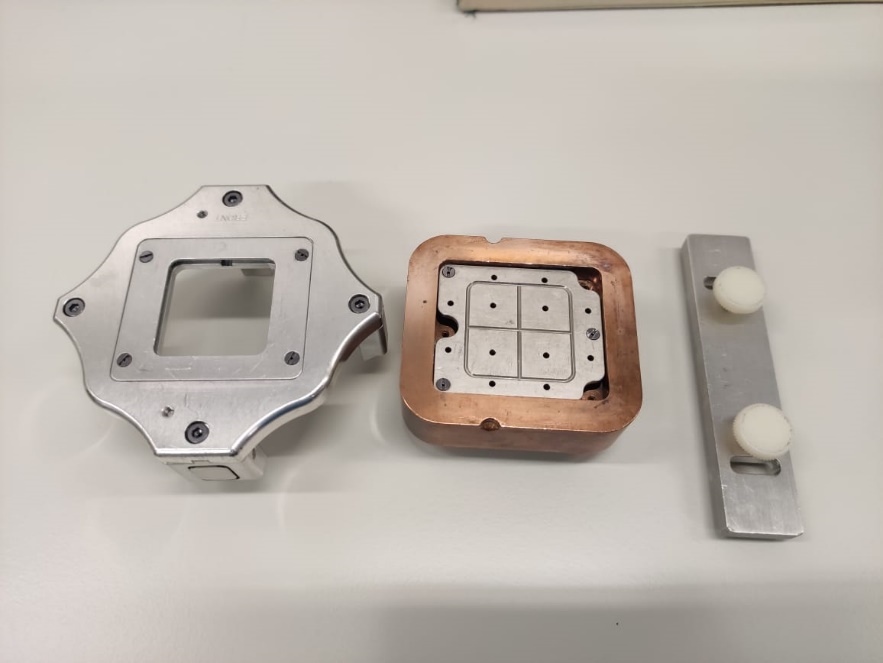

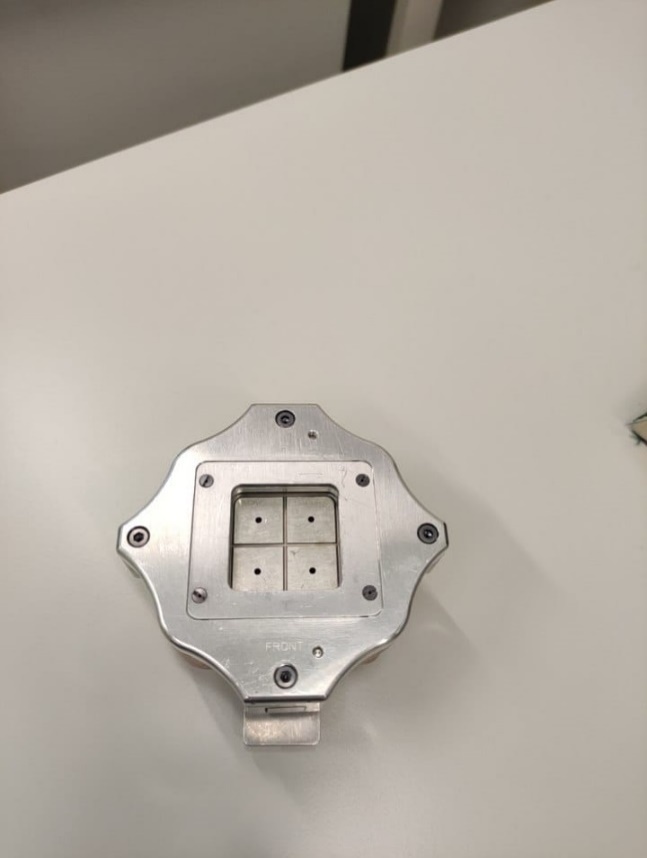


**Supplementary Fig. 30. Photographs of the inert XPS sample holder for the Thermo Fischer K-Alpha XPS system.**

**Supplementary Fig. 31. Ion chromatography calibration curves for NH_4_^+^ in water, 0.002 M LiTFSI and 0.005 M LiTFSI in water.**

**Supplementary Fig. 32. Ion chromatographs of different NH_4_^+^ concentrations in (a) ultrapure water and (b) 0.002 M LiTFSI in water for calibration.**

##

Supplementary Tables

**Supplementary Table. 1. Experimental and performance data from this work and other literature reports in the Li-NRR field.**

| **Ref** | **Mode** | **RE** | ***E*_we_ (V)** | ***E*_Cell_**  **(V)** | ***j***  **(mA cm^-2^)** | ***Q***  **(C)** | **FE**  **(%)** | **Total NH_3_**  **(mmol)** | ***R*_NH3_**  **(nmol s^-1^ cm^-2^)** | **EE_pseudo_**  **(%)** | **EE_batch_**  **(%)** | **EE_continuous_**  **(%)** | ***t***  **(h)** | **Proton**  **Donor** | ***C*_EtOH_**  **(M)** | **Salt** | ***C*_salt_**  **(M)** | ***P*_N2_**  **(bar)** | **System** |
| --- | --- | --- | --- | --- | --- | --- | --- | --- | --- | --- | --- | --- | --- | --- | --- | --- | --- | --- | --- |
| ^11^ | CP | - | - | 30.2 | 25 | 7 | 35±5 | 0.0087±0.0014 | 30±5 | 1.5 | - | 1.3±0.2 ^a^ | 0.08 | EtOH | 0.1 | LiBF_4_ | 1 | 1 | Continuous |
| ^11^ | CP | - | - | 20.3 | 15 | 7.2 | 47.5 | 0.0118±0.0001 | 24.6±2.1 | 2.8 | - | 2.5 | 0.13 | EtOH | 0.1 | LiBF_4_ | 1 | 1 | Continuous |
| ^12^ | CP | Pt | -3.6 V vs. Pt | 4.3 | 6 | 700 | 67±2 | 1.62±0.033 ^a^ | 7.2±0.15 ^a^ | 18±0.7 | - | 14±1 | 2.5 | EtOH | 0.04 | LiBF_4_ | 1 | 1 | Continuous |
| ^14^ | CP | Pt | -3 V vs. Pt | 4 | 6 | 123388 | 64.4 | 274.72 ^a^ | 10.19 | 18.9 | - | 17 | 300 | EtOH | 0.04 | LiBF_4_ | 1 | 1 | Continuous |
| ^13^ | CP | Pt | -3.6 V vs. Pt | 4.3 | 6 | 700 | 72±3 | 1.736±0.05 ^a^ | 7.72±0.22 ^a^ | 19.6±0.8 ^a^ | - | 13.9±0.6 ^a^ | 2.5 | PhOH | 0.037 | LiBF_4_ | 1 | 1 | Continuous |
| ^4^ | CA | - | - | 3.6 | 1 ^b^ | 14.4 ^a^ | 8.9±1.7 | 0.163±0.02 | 0.67±0.04 | 2.9±0.6 | - | 1.9±0.37 | 4 | EtOH | 0.08 | LiBF_4_/PEO | - | 1 | Continuous |
| ^8^ | CP | Pt | - | 10.9 | 1000 | 240 | 71±3 | 0.587 | 2500±100 | 7.7±0.3 | 6.2±0.3 | - | 0.3 | EtOH | 0.17 | LiBF_4_ | 2 | 20 | Batch |
| ^8^ | CP | Pt | - | 6.3 | 1000 | 240 | 45±3 | 0.376 | 1717±110 ^a^ | 8.4±0.6 | 6.0±0.4 | - | 0.3 | EtOH | 0.17 | LiPF_6_ | 2 | 20 | Batch |
| ^8^ | CP | Pt | - | 9.6 | 1000 | 240 | 31±3 | 0.258 | 1149±98 ^a^ | 3.8±0.4 | 3.0±0.3 | - | 0.3 | EtOH | 0.17 | LiClO_4_ | 2 | 20 | Batch |
| ^7^ | CP | Pt | -4.5 V vs. Fc/Fc^+^ | 7 ^b^ | 4 | 150 | 78±1.3 | 0.136±0.002 ^a^ | 10.8±0.2 | 11.7±0.5 | 9.6±0.2 | - | 1.93 | EtOH | 0.17 | LiClO_4_ | 0.3 | 20 | Batch |
| ^9^ | CP | Pt | -0.8 V vs. Li ^b^ | 7 ^b^ | 100 | 50 | 13.3±2 | 0.069±0.009 ^a^ | 46±7 | 2.3±0.3 | 1.6±0.3 | - | 0.8 | EtOH | 0.17 | LiClO_4_ | 2 | 20 | Batch |
| ^6^ | CA | Ag | -0.55 V vs. Li | 6.4 ^b^ | 64 ^b^ | 1122 ^c^ | 99±2 | 3.9±0.2 | 223±8 | 18.4 ^b^ | 13.1±0.3 ^b^ | - | 96 | EtOH | 0.1 | LiTFSI | 2 | 15 | Batch |
| ^15^ | CA | Ag | -0.75 V vs. Li | - | 29.5±10 ^b^ | 2.1±0.4 | 69±7 | 0.005±0.0012 | 58±14 | - | - | - | 2 | Phos | 0.1 | LiBF_4_ | 0.2 | 20 | Batch |
| ^15^ | CP | Ag | - | - | 22.5 | 19.4 | 68.6±0.9 | 0.046±0.006 | 53±2 | - | - | - | 20 | Phos | 0.1 | LiBF_4_ | 0.2 | 20 | Batch |
| ^16^ | CA | Ag | -0.55 V vs. Li | - | 155±4.6 ^b^ | 670±20 | 98±2 | 2.290±0.070 | 530±20 | - | - | - | 24 | EtOH | 0.1 | LiTFSI | 2 | 15 | Batch |
| ^16^ | CA | Ag | -0.55 V vs. Li | - | 137±6.8 ^b^ | 591±29 | 96±1 | 1.843±0.123 | 430±20 | - | - | - | 24 | i-PrOH | 0.1 | LiTFSI | 2 | 15 | Batch |
| ^3^ | CP | - | - | 9 | 1.98 | 20 | 7.5±1.1 | 0.0052±0.001 ^a^ | 0.2±0.03 ^a^ | 1.0±0.1 | 0.7±0.1 | - | 2.8 | EtOH | 0.17 | LiClO_4_ | 0.2 | 1 | Batch |
| ^5^ | CP | Pt | -1 V vs. Li/Li^+^ | 6 ^a^ | 2 | 100 | 37.1±1.3 | 0.130±0.007 ^a^ | 0.42±0.03 ^a^ | 6.9±0.5 | 5.1±0.2 | - | 50 | EtOH | 0.17 | LiClO_4_ | 0.2 | 10 | Batch |
| ^2^ | CP | Pt | - | 15 | 8 | 7 | 19±3 | 0.013±0.002 ^a^ | 5.1±0.8 | 1.5 | 1.3±0.2 | - | 0.7 | EtOH | 0.1 | LiBF_4_ | 1 | 1 | Batch |
| ^17^ | CP | Ag | -3.5 V vs. Li/Li ^b^ | - | 10 | 5 | 39.5±1.7 | 0.0065 | 0.25 ug s^-1^ cm^-2^ | 2.8 | - | - | 0.14 | EtOH | 0.11 | LiBF_4_ | 1 | 1 | Batch |
| ^10^ | CP | Pt | -5.5 V vs. Pt ^b^ | 10 | 4 | 14.4 ^a^ | 45.9±5.6 | 0.0228±0.002 ^a^ | 6.34±0.77 ^a^ | 5.4±0.7 ^a^ | 4.3±0.5 | - | 1 | EtOH | 0.17 | LiClO_4_ | 0.5 | 20 | Batch |
| ^18^ | CP | Ag/AgCl LF | -0.1 V vs. Li/Li^+ b^ | - | 0.004 | 10 | 23.7 | 0.008 ^a^ | 3.27 ^a^ | - | - | - | 0.69 | EtOH | 0.17 | LiFSI | 7 | 1 | Batch |

**Supplementary Table. 1 (Continuous). Experimental and performance data from this work and other literature reports in the Li-NRR field.**

| **Ref** | **Mode** | **RE** | ***E*_we_ (V)** | ***E*_Cell_**  **(V)** | ***j***  **(mA cm^-2^)** | ***Q***  **(C)** | **FE**  **(%)** | **Total NH_3_**  **(mmol)** | ***R*_NH3_**  **(nmol s^-1^ cm^-2^)** | **EE_pseudo_**  **(%)** | **EE_batch_**  **(%)** | **EE_continuous_**  **(%)** | ***t***  **(h)** | **Proton**  **Donor** | ***C*_EtOH_**  **(M)** | **Salt** | ***C*_salt_**  **(M)** | ***P*_N2_**  **(bar)** | **System** |
| --- | --- | --- | --- | --- | --- | --- | --- | --- | --- | --- | --- | --- | --- | --- | --- | --- | --- | --- | --- |
| Own work | CA | LFP | -3.2 V vs. SHE | 5 | 22.8 | 32.8 | 19.7 | 0.022 | 15.5 | 4.57 | 3.1 | - | 4 | EtOH | 0.1 | LiTFSI | 2 | 20 | Batch |
| Own work | CA | LFP | -3.7 V vs. SHE | 6.7 | 184.2±6.0 | 265.3±8.6 | 48.0±3.9 | 0.44±0.05 | 306.3±35.0 | 8.4±0.7 | 6.2±0.5 | - | 4 | EtOH | 0.1 | LiTFSI | 2 | 20 | Batch |
| Own work | CA | LFP | -4.6 V vs. SHE | 7.2 | 94.1±6.6 | 135.5±9.5 | 62.9±2.2 | 0.29±0.01 | 203.9±7.3 | 10.2±0.4 | 7.6±0.3 | - | 4 | EtOH | 0.1 | LiTFSI | 2 | 20 | Batch |

^a^ Recalculated based on other data mentioned in the publication.

^b^ Derived from graph in the literature entree.

**Supplementary Table. 2. Respective composition ratios of identified Li- and F-compounds in the SEI determined by ssNMR.** This data is supporting Figure 7a and 7b from the main manuscript.

| *E*_we_ | ^7^Li NMR | | ^19^F NMR | | |  |
| --- | --- | --- | --- | --- | --- | --- |
| V vs SHE | Metallic Li | SEI/LiTFSI | | LiTFSI | LiF | |
| -3.7 | 1±1% | 99±1% | | 45±8% | 55±8% | |
| -4.6 | 37% | 63% | | 26% | 74% | |

## Supplementary Discussion

**S1. Liquid NMR analysis**

The ^1^H NMR spectra of 2 M LiTFSI in 0.1 M EtOH/THF shows two large THF solvent peaks at 3.77 ppm and 1.89 ppm (including small satellites) and EtOH peaks at 1.20 ppm (t, -CH_3_) and 4.81 ppm (t, -OH). The EtOH peak (m) associated with -CH_2_- is not observable and most likely overlaps with THF. We also observed a sharp peak at 4.64 ppm (s), which is most likely water left-over in the NMR tube after the cleaning procedure. The peaks at 2.67 ppm (s) and 6.97 ppm (s) are associated with the DMSO as internal standard and C_6_H_6_ from the C_6_D_6_ locking solvent. ^13^C NMR was executed in complementary with 1H NMR, where the spectra of 2 M LiTFSI in 0.1 M EtOH/THF showed two THF peaks at 26.24 ppm and 68.67 ppm and two EtOH peaks at 18.30 ppm and 58.79 ppm. The downfield peaks forming a quartet at 116.13 ppm, 119.13 ppm, 122.54 ppm and 125.71 ppm represent the -CF_3_ functionality from TFSI. At last, the peak (t) at 128.62 ppm and 40.48 ppm are signals from the locking solvent (C_6_H_6_) and DMSO as internal standard.

After applying *E*_we_ ≤ -3.7 V, the NMR analysis of the electrolyte (post measurement) shows a significant number of new peaks. In the ^1^H NMR spectra, a new peak starts to appear at 1.13 ppm (t) next to the -CH_3_ functionality of EtOH, which could indicate that one of the THF oxidation side products preserves an ethoxy group. The ^13^C NMR spectra shows six new peaks at 15.57 ppm, 24.13 ppm, 32.93 ppm, 63.08 ppm, 67.36 ppm and 104.49 ppm that match well with the reference spectrum of 2-ethoxy-tetrahydrofuran as was previously identified by MacFarlane, Simonov and coworkers. Other signals close to the C-C-C (27.40 ppm) and O-C-C (71.31 ppm) bond environment of THF may indicate the presence of polymerized THF chains. This suggests that there are multiple stable side-products formed during solvent oxidation, which could indicate that the THF^+^ cation intermediate does not preferentially react with EtOH but can also initiate ring-opening THF polymerization. Both polymerized THF and 2-ethoxy-tetrahydrofuran were not detected as side products after low overpotential measurements (-3.2 V), which suggests that the solvent oxidation mechanism is to some extent dependent on the anodic potential. While other peaks were present in the ^13^C NMR spectrum, it was not possible to allocate them to certain side products.

**S2. Energy efficiency calculation**

The general expression for the energy efficiency of an ammonia production process is defined as the ratio between the energy content of the ammonia product stream (*e*_out_) and the sum over all the energy input contributions (Σ*e*_in_) from the process units, such as feed gas pretreatment, power demand of the electrolyzer, separation/purification and storage.^19^ For simplicity, we only consider the energy input associated with the electrolyzer unit.

$$\mathrm{EE}=\frac{e_{\mathrm{out}}}{\sum e_{\mathrm{in}}}$$

The lower heating value of ammonia (18.6 kJ g^-1^) is typically used in the chemical industry as *e*_out_, while the gibbs free energy of the ammonia oxidation reaction (Δ*G*_NH3_ = 19.9 kJ g^-1^) appears to be more common in the scientific literature.^2, 12^ We used the latter for comparison purposes which allowed us to express *e*_out_ as the standard equilibrium potential of ammonia oxidation ($E_{NH3}^{0}$):

$$e_{\mathrm{out}}=n_{NH3}*\Delta G_{NH3}=n_{NH3}*\eta_{NH3}*F*E_{NH3}^{0}$$

Where, n_NH3_ is the total amount of moles NH_3_ produced, *η*_NH3_ the number of electron-transfer step to produce ammonia (*η*_NH3_ = 3), and *F* is the Faraday constant (*F* = 96485 C mol^-1^). Manthiram and co-workers used the total cell voltage (*E*_cell_) measured by the potentiostat as *e*_in_, however the energy loss from the sacrificial solvent oxidation (batch cell) or associated with hydrogen production (continuous flow cell) was not included. Chorkendorff and coworkers coined this term the pseudo EE and was re-written in a voltage-Faradaic efficiency (FE_NH3_) relationship:^12^

$$EE_{\mathrm{pseudo}}=\frac{n_{NH3}*\Delta G_{NH3}}{E_{\mathrm{cell}}*Q_{\mathrm{total}}}= \frac{n_{NH3}*\eta_{\mathrm{NRR}}*F*E_{NH3}^{0}}{E_{\mathrm{cell}}*Q_{\mathrm{total}}}=\frac{n_{NH3}*\eta_{NH3}*F*E_{NH3}^{0}}{E_{\mathrm{cell}}*Q_{NH3}}FE_{NH3}$$

$$=\frac{n_{NH3}*\eta_{NH3}*F*E_{NH3}^{0}}{E_{\mathrm{cell}}*\eta_{NH3}*F*n_{NH3}}FE_{NH3}=\frac{E_{NH3}^{0}}{E_{\mathrm{cell}}}FE_{NH3}$$

We further extend the EE_pseudo_ by including the energy loss from the sacrificial solvent. For simplification, we only consider THF as being consumed by oxidation. The energy requirement to produce THF via an industrial process plant was taken from Lange et al. and used as an additional energy input (*e*_THF_ = 6.6 kJ g^-1^).^20^ The amount of THF that is consumed by the THF oxidation reaction can be expressed in the more useful expression of the FE_NH3_ by assuming *Q*_THF_ ≈ *Q*_total_, since THF oxidation is the main oxidation reaction in our system.

$$n_{THF}=\frac{Q_{THF}}{\eta_{THF}F}=\frac{Q_{total}}{\eta_{THF}*F}=\frac{Q_{NH3}}{\eta_{THF}*F*FE_{NH3}}$$

The full derivation of the EE for a Li-NRR batch cell becomes:

$$EE_{\mathrm{batch}}=\frac{n_{NH3}*\Delta G_{NH3,ox}}{n_{\mathrm{THF}}*e_{\mathrm{THF}}+E_{\mathrm{cell}}*Q_{\mathrm{total}}}= \frac{n_{NH3}*\eta_{NH3}*F*E_{NH3}^{0}}{e_{\mathrm{THF}}*\frac{Q_{\mathrm{THF}}}{\eta_{\mathrm{THF}}F}+E_{\mathrm{cell}}*\frac{Q_{NH3}}{FE_{NH3}}}=\frac{n_{NH3}*\eta_{NH3}*F*E_{NH3}^{0}}{e_{\mathrm{THF}}*\frac{Q_{NH3}}{\eta_{\mathrm{THF}}*F*FE_{NH3}}+E_{\mathrm{cell}}*\frac{Q_{NH3}}{FE_{NH3}}}=\frac{n_{NH3}*\eta_{NH3}*F*E_{NH3}^{0}}{\left( \frac{e_{THF}}{\eta_{THF}*F}+E_{cell} \right)}\frac{FE_{NH3}}{Q_{NH3}}=\frac{n_{NH3}*\eta_{NH3}*F*E_{NH3}^{0}}{\left( \frac{e_{\mathrm{THF}}}{\eta_{\mathrm{THF}}*F}+E_{\mathrm{cell}} \right)}\frac{FE_{NH3}}{\eta_{NH3}*F*n_{NH3}}=\frac{E_{NH3}^{0}}{\left( \frac{e_{\mathrm{THF}}}{\eta_{\mathrm{THF}}*F}+E_{\mathrm{cell}} \right)}FE_{NH3}$$

The EE of a continuous cell with hydrogen oxidation and the energy input from a water splitting electrolyzer ($E_{H2}^{0}$ = 1.23 V and EE_H2_ = 70%) was taken directly from Fu et al.:^12^

$$EE_{\mathrm{continuous}}=\frac{E_{NH3}^{0}}{\left( \frac{E_{H2}^{0}}{EE_{H2}}+E_{\mathrm{cell}} \right)}FE_{NH3}$$

To estimate the maximum EE of our system using EE_pseudo_ and EE_batch_, we use the equilibrium potentials of Li plating (-3.02 V vs. SHE) and THF oxidation (+1 V vs. SHE) as the cell voltage (*E*_cell_ = 4.02 V) and assume a FE of 100%. For the continuous process, the minimum cell voltage is 3.02 V due to combination of the equilibrium potential of Li plating (-3.02 V vs. SHE) and hydrogen oxidation (0 V vs. SHE). The maximum EE for the different systems are listed below:

$$EE_{pseudo,max}=\frac{1.17}{4.02}*100\%=29\%$$

$$EE_{batch,max}=\frac{1.17}{\frac{475000}{2*96485}+4.02}*100\%=18\%$$

$$EE_{continuous,max}=\frac{1.17}{\frac{1.23}{0.7}+3.02}*100\%=25\%$$

## Supplementary References

1. Hoffmann GG. *Infrared and Raman Spectroscopy: Principles and Applications*. Walter de Gruyter GmbH & Co KG (2023).

2. Lazouski N, Schiffer ZJ, Williams K, Manthiram K. Understanding Continuous Lithium-Mediated Electrochemical Nitrogen Reduction. *Joule* **3**, 1127-1139 (2019).

3. Andersen SZ*, et al.* A rigorous electrochemical ammonia synthesis protocol with quantitative isotope measurements. *Nature* **570**, 504-508 (2019).

4. Cai X*, et al.* Membrane electrode assembly design for lithium-mediated electrochemical nitrogen reduction. *Energy & Environmental Science* **16**, 3063-3073 (2023).

5. Andersen SZ*, et al.* Increasing stability, efficiency, and fundamental understanding of lithium-mediated electrochemical nitrogen reduction. *Energy & Environmental Science* **13**, 4291-4300 (2020).

6. Du H-L*,* Chatti M, Hodgetts RY, Cherepanov PV, Nguyen CK, Matuszek K, MacFarlane DR, Simonov AN. Electroreduction of nitrogen with almost 100% current-to-ammonia efficiency. *Nature 2022 609:7928* **609**, 722-727 (2022).

7. Li K*, et al.* Enhancement of lithium-mediated ammonia synthesis by addition of oxygen. *Science* **374**, 1593-1597 (2021).

8. Li S*, et al.* Electrosynthesis of ammonia with high selectivity and high rates via engineering of the solid-electrolyte interphase Electrosynthesis of ammonia with high selectivity and high rates via engineering. *Joule* **6**, 2083-2101 (2022).

9. Li K*, et al.* Increasing Current Density of Li-Mediated Ammonia Synthesis with High Surface Area Copper Electrodes. *ACS Energy Letters* **7**, 36-41 (2022).

10. Lim C*,* Kim D, Kim M, Yun H, Shin D, Hwang YJ, Shin H, Yong K. Effect of Sulfur-Derived Solid Electrolyte Interphase on Li-mediated Nitrogen Reduction. *ACS Energy Letters* **8**, 4875-4884 (2023).

11. Lazouski N, Chung M, Williams K, Gala ML, Manthiram K. Nonaqueous gas diffusion electrodes for rapid ammonia synthesis from nitrogen and water. *Nature Catalysis* **3**, 463-469 (2020).

12. Fu X*, et al.* Continuous-flow electrosynthesis of ammonia by nitrogen reduction and hydrogen oxidation. *Science* **379**, 707-712 (2023).

13. Fu X*, et al.* Phenol as proton shuttle and buffer for lithium-mediated ammonia electrosynthesis. *Nature Communications* **15**, 2417 (2024).

14. Li S*, et al.* Long-term continuous ammonia electrosynthesis. *Nature* **629**, 92-97 (2024).

15. Suryanto BHR*,* Matuszek K, Choi J, Hodgetts RY, Du H-L, Bakker JM, Kang CSM, Cherepanov PV, Simonov AN, MacFarlane DR. Nitrogen reduction to ammonia at high efficiency and rates based on a phosphonium proton shuttle. *Science* **372**, 1187-1191 (2021).

16. Du H-L*,* Matuszek K, Hodgetts RY, Dinh KN, Cherepanov PV, Bakker JM, MacFarlane DR, Simonov AN. The chemistry of proton carriers in high-performance lithium-mediated ammonia electrosynthesis. *Energy & Environmental Science* **16**, 1082-1090 (2023).

17. Cai X*,* Fu C, Iriawan H, Yang F, Wu A, Luo L, Shen S, Wei G, Shao-Horn Y, Zhang J. Lithium-mediated electrochemical nitrogen reduction: Mechanistic insights to enhance performance. *Iscience* **24**, 103105 (2021).

18. Iriawan H, Herzog A, Yu S, Ceribelli N, Shao-Horn Y. Upshifting Lithium Plating Potential To Enhance Electrochemical Lithium Mediated Ammonia Synthesis. *ACS Energy Letters* **9**, 4883-4891 (2024).

19. Izelaar B*,* Ramdin M, Vlierboom A, Pérez-Fortes M, Van der Slikke D, Kumar AS, De Jong W, Mulder FM, Kortlever R. Techno-economic assessment of different small-scale electrochemical NH_3_ production plants. *Energy & Environmental Science* **17**, 7983-7998 (2024).

20. Lange JP, Wadman SH. Furfural to 1, 4‐Butanediol/Tetrahydrofuran–A Detailed Catalyst and Process Design. *ChemSusChem* **13**, 5329-5337 (2020).
